# Social connectedness and suicidal ideation: the roles of perceived burdensomeness and thwarted belongingness in the distress to suicidal ideation pathway

**DOI:** 10.1186/s40359-023-01338-5

**Published:** 2023-10-06

**Authors:** Peter Richard Gill, Michael Arena, Christopher Rainbow, Warwick Hosking, Kim M Shearson, Gavin Ivey, Jenny Sharples

**Affiliations:** 1 Institute for Health and Sport, Victoria University, Footscray Park, Ballarat Rd, Melbourne, VIC Australia; 2 Centre for Social and Early Emotional Development (SEED), School of Psychology, Deakin University, Melbourne, Australia

**Keywords:** Suicide, Suicidal ideation, Distress, Burdensomeness, Belongingness, Social connectedness

## Abstract

**Background:**

Suicide is a serious public health issue. Distress has been identified as a common risk factor, with research also suggesting that a lack of social connectedness is involved.

**Methods:**

This quantitative, cross-sectional study investigated the role of perceived burdensomeness and thwarted belongingness in the psychological distress/suicidal ideation pathway in a community sample of 480 Australian adults.

**Results:**

As expected, distress was found to be a strong predictor of suicidal ideation. Perceived burdensomeness and thwarted belongingness both moderated and mediated the relationship between distress and suicidal ideation. Specifically, distress was more strongly linked to suicide ideation when burdensomeness or thwarted belongingness were also high. This moderating effect was stronger for thwarted belongingness than it was for burdensomeness. These variables also mediated the pathway, in that higher distress related to higher burdensomeness and thwarted belonging, which in turn related to higher suicide ideation. This mediating effect was stronger for burdensomeness than for thwarted belonging.

**Conclusions:**

Overall, the findings confirm the importance of our social relatedness in suicide. Increasing belongingness and reducing the perception of being a burden on others may be an important intervention strategy for weakening the link between distress and suicide ideation.

## Introduction

Suicide is a serious public health issue worldwide, with over 700,000 deaths recorded annually. [1] In Australia, suicide is the leading cause of death for young adults, with rates among this group rising over the past decade [2]. A meta-analysis examining 50 years of research has concluded that many currently known risk factors for suicide have a predictive value little better than chance, [3] highlighting the importance of early intervention at the point where suicidal thoughts first emerge. While suicidal thoughts do not always lead to attempts or fatalities, [4] the prolonged presence of suicidal thoughts over time can increase the risk of death by suicide, [5] and are associated with a declining mental and physical health trajectory over multiple years [6]. It has been proposed that universal screening for suicidal thoughts in healthcare settings is a feasible method of detecting people at risk of suicide, [7] however once detected it is crucial to gain an understanding of the psychosocial drivers underlying the suicidal thoughts, so that a treatment plan can be devised to directly address them [8].

Psychological distress, a state of emotional suffering often characterised by depressive and anxious symptoms, [9] has been strongly linked with suicidal thoughts [10, 11]. In Australia, high levels of psychological distress are experienced by at least 1 in 7 people in a four-week period, with rates higher in young adults [12]. However, psychological distress alone does not inevitably lead to the development of suicidal thoughts [13]. The integrated motivational-volitional (IMV) model of suicidal behaviour proposes a motivational phase that can follow elevated levels of distress, a phase which includes feelings of defeat, humiliation, and entrapment as precursors to the development of suicidal thoughts [14]. Whether suicidal thinking develops from these feelings is dependent on the presence of several ‘motivational moderators’ which can strengthen or weaken suicidal intent [14]. These include belongingness, a feeling of being connected to others in mutually satisfying and reciprocal relationships [15]; and perceived burdensomeness, a set of beliefs that one’s presence is a drain on other people and society, leading to thoughts of suicide as a beneficial solution [15].

The concepts of thwarted belonging (TB) and perceived burdensomeness (PB) are also central components in the interpersonal-psychological theory of suicidal behaviour (IPS), which proposes that both these conditions interact to produce suicidal thinking, along with a sense of hopelessness about the possibility of change [15]. There have been three systematic reviews of the IPS processes on suicidal thoughts and behaviours, [16–18] producing mixed results. The review by Ma et al. [16] concluded that PB is more often significantly related to suicidal ideation than TB (significance reached in 83% of studies for PB vs. 40% of studies for TB), while noting that TB has been less studied. The meta-analysis conducted by Chu et al. [17] encompassing 46 studies found that the interaction of TB and PB on suicidal ideation was significant, but modest (*r* = .14), with stronger effects being found separately for univariate associations between suicidal ideation and TB (*r* = .37) and PB (*r* = .48). The final review by Espinosa-Salido et al. [18] focused on the indirect effects of TB and PB, concluding that they each acted as mediators between aspects of psychological distress and suicidal thinking, but again more strongly as individual constructs rather than interacting ones.

The case for examining TB and PB separately makes theoretical as well as practical sense. For example, it is possible to feel a deep sense of connection to other people while simultaneously believing oneself to be a burden on those others, [19] or to have few close relationships where beliefs of perceived burden could be attached [20]. The relatively smaller number of TB studies compared to PB also leaves an important gap in literature, given that the risk of death by suicide is known to decrease as the number of social connections increases [21]. The concept of TB encompasses several aspects of social connectedness, including strength and quality of perceived interpersonal relationships, and frequency of interaction with others. [22] Given that social connectedness is a known protective factor against suicidal ideation, [23] further research is warranted to explore the role of TB in bridging and intensifying the relationship between psychological distress and suicidal ideation.

Worst-point suicidal ideation is predictive of the transition to suicide attempts and fatalities, [24, 25] so understanding how psychosocial factors work to intensify suicidal ideation may additionally help in the development of better targeted and more timely intervention efforts. While a number of studies have explored the role of TB and PB as mediators, [16, 18] moderating effects of TB and PB on the relationship between psychological distress and suicidal ideation appear less explored, presenting a crucial gap from a suicide risk perspective. Experience sampling studies have shown that psychological distress and suicidal ideation can intensify dramatically over the course of a few hours, [26, 27] with interpersonal negative life events predicting greater intensity of next-hour suicidal thoughts [28]. This potentially rapid increase in risk illustrates the importance of exploring how TB and PB may intensify the relationship between distress and suicidal thought processes.

**Fig. 1:**
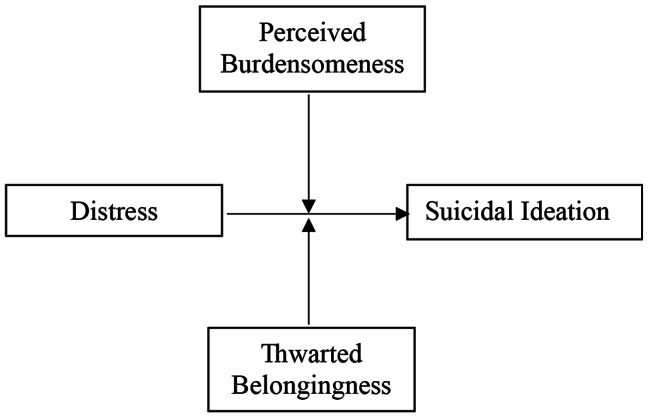
Moderating effect of PB and TB on the Relationship between Psychological Distress and Suicide Ideation (separate analysis for each moderator)

**Fig. 2:**
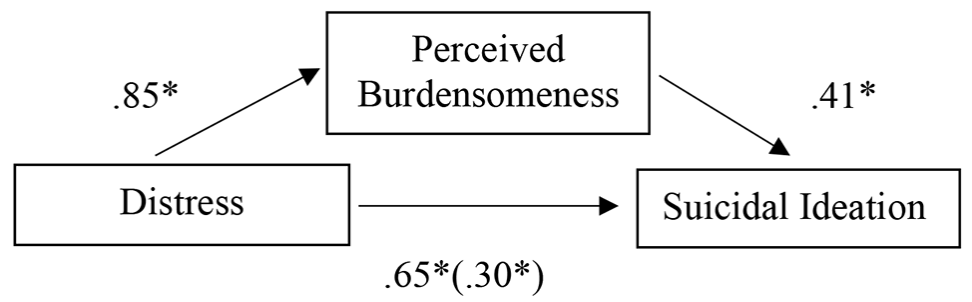
Regression coefficients for the relationship between distress and suicide ideation as mediated by PB

**Fig. 3:**
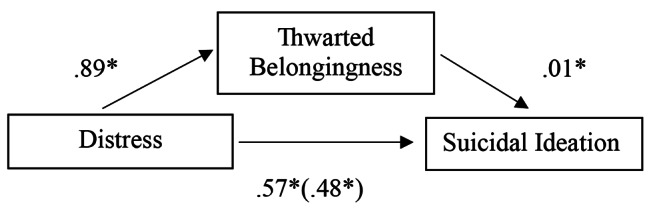
Regression coefficients for the relationship between distress and suicide ideation as mediated by TB

To facilitate the development of more targeted interventions for suicidal ideation in the future, the present study therefore aimed to examine separately the mediating and moderating influence of TB and PB on suicidal ideation, using a community-based sample of Australian adults. It was hypothesised that:

1. Higher levels of psychological distress will be associated with higher levels of suicide ideation;
2. PB will moderate the relationship between psychological distress and suicidal ideation such that higher levels of PB will be associated with higher levels of psychological distress and suicidal ideation (Fig. 1);
3. TB will moderate the relationship between psychological distress and suicidal ideation such that higher levels of TB will be associated with higher levels of psychological distress and suicidal ideation (Fig. 1);
4. PB will mediate psychological distress and suicide ideation (Fig. 2), with increased distress leading to increased feelings of PB which in turn increase suicide ideation; and.
5. TB will mediate psychological distress and suicide ideation (Fig. 3) with increased distress leading to an increase in feelings of TB which in turn increases suicide ideation.

## Method

### Participants

Participants were recruited through Prolific, a paid online survey recruitment platform. International studies on the demographic representation of online samples for statistical studies demonstrate high validity for generating representative population samples [29]. Prolific participant responses were screened and deleted where unusually brief or patterned responses occurred. Participants were required to be 18 years of age or older, and reside in Australia. As the sample required a general representation of the Australian population, a history of mental distress, mental illness, or suicidality was not required to participate. No other inclusion or exclusion criteria were applied. Participants were paid 15AUD per hour (pro rata) for their time taking the survey. Cases with missing data were deleted (n = 16). Data from 480 participants with an average age of 33.7 (range = 18–75 years old) were analysed for this study (Table 1).

**Table 1:** Demographic data of participants (n = 480)

Variable		*n (%)*
Gender		
	Male	276 (57.5)
	Female	200 (41.7)
	Non-binary / third gender	4 (0.8)
Sexual Orientation		
	Heterosexual	380 (79.2)
	Gay	27 (5.6)
	Bisexual	53 (11.0)
	Prefer not to say	7 (1.5)
Relationship Status		
	Married	133 (27.7)
	Widowed Divorced	2 (0.4) 17 (3.5)
	De facto (living with partner)	110 (22.9)
	Separated	6 (1.3)
	Single	212 (44.2)
Location		
	Metropolitan	384 (80.0)
	Rural	94 (19.6)
	Remote	2 (0.4)
Financial Ability to Meet Basic Needs		
	I struggle to meet my daily needs	32 (6.7)
	I can afford to meet my daily needs but not much else	257 (53.5)
	I can afford my daily needs and the luxuries in life	191 (39.8)

### Measures

#### Suicidal ideation attributes scale (SIDAS)

The SIDAS is a five-item scale assessing frequency of SI (item 1), controllability of ideations (item 2), closeness to an attempt (item 3), level of distress (item 4), and interference with daily activities (item 5) over the past month rated on 10-point scales [30]. Total SIDAS scores are calculated as the sum of the five items, with controllability (item 2) reverse scored. Total scale scores range from 0 to 50. Higher scores are indicative of greater suicidal ideation severity. The internal consistency for the current study was C_α_=0.80.

#### Kessler Psychological Distress Scale (K-10)

K-10 is a 10-item questionnaire assessing general psychological distress in the 30 days prior to administration [31]. Items assess symptoms commonly associated with depressive and anxiety disorders. Responses are scored on a five-point scale from 0 to 4, with increasing values corresponding to higher levels of distress. A total score ranging from 10 to 50 is derived by summing all items. The Cronbach alpha for the current study was C_α_=0.93.

#### Interpersonal needs questionnaire (INQ-15)

The Interpersonal Needs Questionnaire is 15-item self-report measure [32]. The INQ-15 includes six questions to measure Perceived Burdensomeness ( item 1 to item 6) and nine questions to assess Thwarted Belongingness. Participants record their responses to the presented statements on a 7-point Likert-type scale, where 1 indicates “Not at all like me”, and 7 indicates “Very true for me”. Items 7, 8, 10, 13, 14, and 15 of the Thwarted Belongingness subscale are reverse-coded. Thus, higher scores reflect higher levels of Thwarted Belongingness and Perceived Burdensomeness. Internal consistency was strong in the current study, PB C_α_=0.96, and TB C_α_=0.91,

### Procedure

Approval was sought and granted for this study by the Victoria University Research Ethics Committee. Data was collected online through the Qualtrics survey platform.

### Design and statistical analysis

The current study was guided by a positivist epistemology and utilised a quantitative, correlational design. Descriptive analyses and moderated regressions were performed using IBM SPSS Statistics version 27 with the PROCESS macro. Moderation and Mediation analysis was tested using PROCESS (Model 1 and 4 respectively). [33] Moderation occurs when the interaction term 95% CI does not contain zero. Mediation occurs when the indirect effect term 95% CI does not contain zero.

## Results

### Analysis

#### Descriptive statistics

Mean and standard deviations for the variables are presented in Table 2.

**Table 2:** Mean scores and standard deviations for the variables

Variable	*n M (SD)*	
SIDAS K10 Perceived Burdensomeness Thwarted Belongingness INQ − 15	478 480 472 402 399	6.12 (8.42) 21.76 (8.32) 7.99 (9.97) 29.71 (12.45) 36.79 (19.40)

An independent sample t-test revealed no significant difference on SIDAS between male and female participants *F*(472) = 0.866, *p* = .352. As a result, the variable of gender was not included in any further analysis. Mean distress was in mild distress range, while suicide ideation was low.

A significant negative relationship was found between age and the variables of distress, ideation, PB and TB, suggesting that distress decreases with age. Distress, ideation, PB and TB were all positively interrelated. The correlations matrix results can be seen in Table 3.

**Table 3:** Correlation Matrix of Age with Psychological Distress, Suicide Ideation, and Interpersonal Needs

	Psychological Distress	Suicide Ideation	Perceived Burdensomeness	Thwarted Belongingness
Age	− 0.268*	− 0.171*	− 0.170*	− 0.133*
Psychological Distress		0.630*	0.708*	0.567*
Suicide Ideation			0.696*	0.443*
Perceived Burdensomeness				0.626*

*<0.001

#### Hierarchical regression for hypothesis testing

To test the hypotheses that PB and TB moderated the relationship between psychological distress and suicide ideation a hierarchical regression model was conducted using PROCESS macro (Model 1) [33]. In line with recommendations by Espinosa-Salido et al., [18] the variables were entered into separate models. All scores were mean centred as part of the analysis.

##### Hypothesis 1

Higher levels of psychological distress will predict higher levels of suicide ideation.

Distress was a significant unique predictor in both moderation models, as seen in Table 4.

**Table 4:** Results of Moderation analysis in PROCESS Procedure for SPSS Version 4.0 for Emotional Distress Predicting Suicide Ideation

Variable		B	SE B	*t*	*p*	LLCI	ULCI
Distress^a^		0.30	0.04	6.79	0.00*	0.21	0.39
PB^a^		0.30	0.04	6.72	0.00*	0.21	0.38
Distress x PB^a^		0.01	0.00	4.61	0.00*	0.01	0.02
Distress^b^		0.40	0.05	8.44	0.00*	0.31	0.50
TB^b^		0.12	0.03	4.11	0.00*	0.06	0.18
Distress x TB^b^		0.02	0.00	5.61	0.00*	0.01	0.02

a. Perceived Burdensomeness b. Thwarted Belongingness *<.001

##### Hypothesis 2

Perceived burdensomeness will moderate the relationship between psychological distress and suicidal ideation.

For the model incorporating PB, distress and SI, there was significant effect with *F*(3,466) = 189.23, *p* < .001, *R*^*2*^ = 0.55 as seen in Table 4. The interaction of psychological distress and PB also showed a significance effect at Δ*R*^*2*^ = 0.02, *F*(1,466) = 21.25, *p* < .001.

**Fig. 4:**
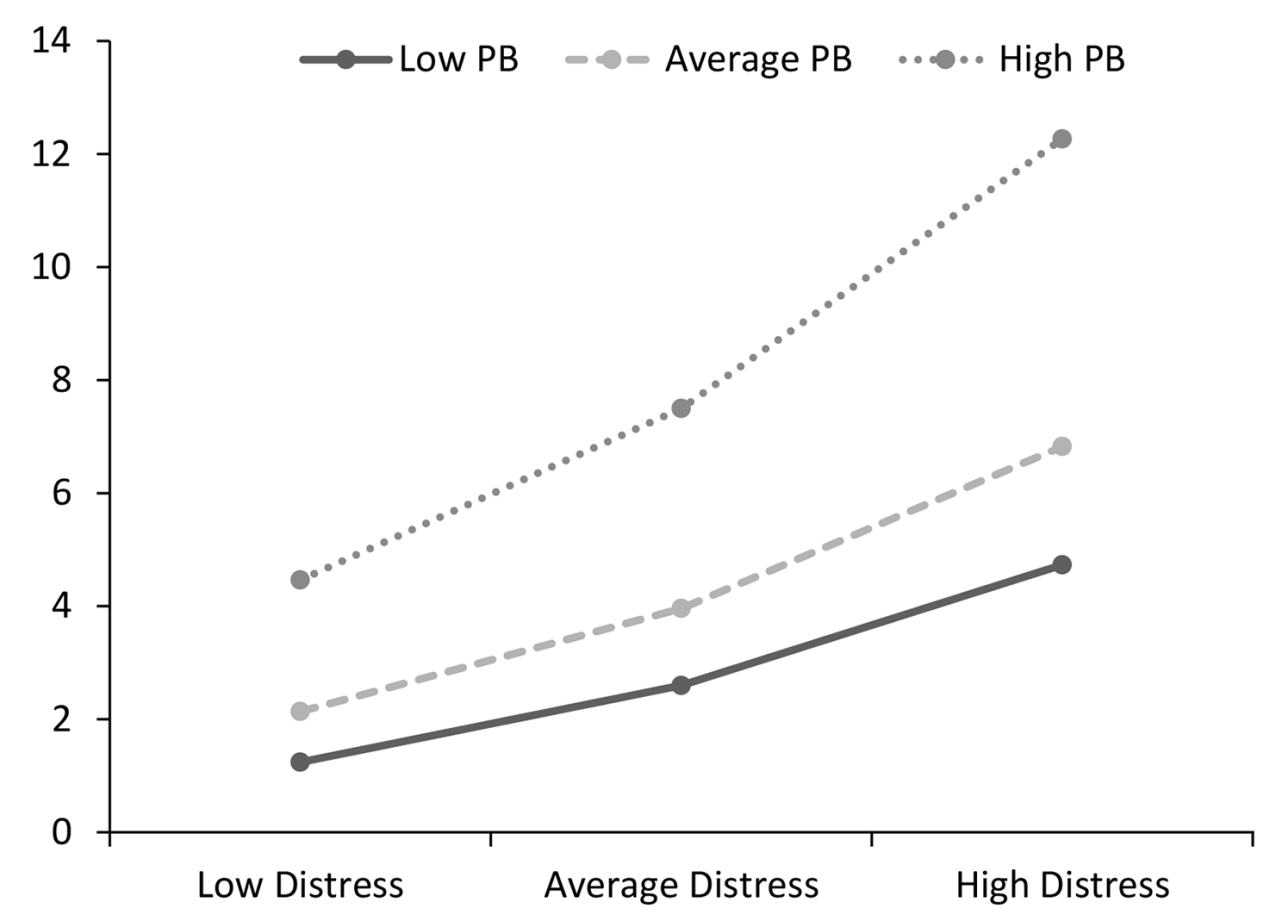
The effect of perceived burdensomeness (PB) on the relationship between psychological distress and suicide ideation. All variables were standardised to mean 0, standard deviation

An analysis of the conditional effects of the focal predictor at values of the moderator revealed as PB increases it strengthens the relationship between distress and suicidal ideation. This effect can be seen in the interaction plot which also showed that high PB is a stronger moderator when present with high distress (Fig. 4).

##### Hypothesis 3

Thwarted Belongingness will moderate the relationship between psychological distress and suicidal ideation.

For the model incorporating TB, psychological distress and suicidal ideation, there was a significant effect (Table 4), with *F*(3,397) = 92.41, *p <* .001, *R*^*2*^ = 0.41. The interaction of psychological distress and TB also showed a significance effect at Δ*R*^*2*^ = 0.05, *F*(1,397) = 31.49, *p <* .001.

**Fig. 5:**
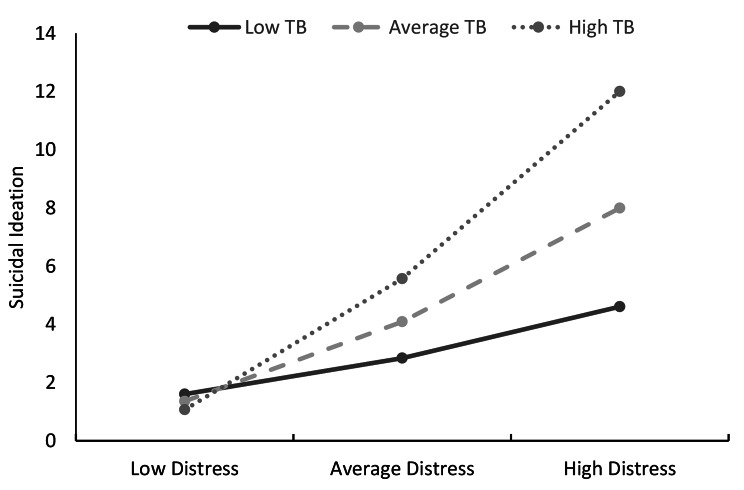
The moderating effect of thwarted belongingness (TB) on the relationship between psychological distress and suicide ideation. All variables were standardised to mean 0, standard deviation ± 1

An analysis of conditional effects of distress at values of TB indicated that the presence of low, medium and high levels of TB were irrelevant when distress was low. Figure 5 displays the enhancing effects of TB on distress showing that the relationship between distress and ideation becomes stronger as TB increases.

##### Hypothesis 4 and Hypothesis 5

Perceived burdensomeness and Thwarted Belongingness Will Mediate Psychological Distress and Suicide Ideation*.*

To test the hypotheses that PB and TB mediated the relationship between psychological distress and suicide ideation an analysis using PROCESS (Model 4) [33] in SPSS was conducted for each of the mediators with 5000 bootstrapped samples. PB was a significant mediator of distress, with an indirect effect of 0.34, 95%CI (0.25, 0.42). The mediating effect of TB was also significant at 0.09, 95%CI (0.04, 0.14), while weaker than PB (see Figs. 4 and 5).

## Discussion

The current study investigated whether the relationship between psychological distress and suicidal ideation was mediated and/or moderated by perceived burdensomeness (PB) and thwarted belongingness (TB). All hypotheses were supported. As expected, distress was positively linked to SI. PB and TB both moderated and mediated the relationship between distress and SI. Specifically, distress was more strongly linked to SI when PB or TB were also high. The moderating effect of TB was stronger than PB, while the mediating effect of PB was stronger than TB (very small mediating effect). These findings are consistent with the ‘motivational moderator’ components of the integrated motivational-volitional theory (IMV) of suicidal behaviour [14], as well as the interpersonal and three-step theories of suicide [15, 23]. The results confirm that, in an Australian community sample, feelings of social disconnection and perceived burden intensify the link between distress and SI. Consistent with earlier research, [34–36] age was negatively associated with distress and SI, but also with TB and PB; in contrast with some studies that have associated PB more with older age [37, 38]. Although Australian population health data has shown that levels of distress and SI are higher in women, [2] no gender differences were found here.

These findings add nuance to previous relationships found between distress, TB, PB and SI. Compared to TB, PB formed a stronger bridge between psychological distress and suicidal ideation as a mediator. As suggested by the ‘differential activation hypothesis,’ [39] high levels of distress may prime feelings of perceived burdensomeness, which in turn lead to thoughts of suicide. According to the IMV model of suicide, such pathways can become reinforced over time, such that each new episode of distress facilitates a quicker transition to thoughts of suicide, via the previously-primed PB. [14] Given the negative associations previously found between PB, SI, and help-seeking intent, [13, 40] such findings have important suicide risk implications for people experiencing high levels of PB, which may hinder support-seeking during times of crisis. Cognitive-behavioural interventions that focus on reducing PB may be effective in disrupting these pathways, [41, 42] allowing people experiencing high levels of distress to question their thought processes and marshal alternate coping resources. While TB was a significant mediator, the effect was small. This is consistent with cross-sectional findings where TB is more weakly related to SI than PB [17]. It has also been suggested that the Interpersonal Needs Questionnaire may not fully capture the TB construct [43].

In contrast to the mediation effects found, TB was a stronger moderator of the distress-SI relationship than PB. High distress in combination with high TB was associated with higher SI, suggesting that participants experiencing greater levels of distress regarding the quality of their interpersonal connections feel more intensely suicidal. This relationship may be bi-directional, with higher levels of distress driving an increased sense of isolation, [44] or social withdrawal acting as an expression of distress. For example, distress may cause reduced interaction with others, leading to increased social isolation and the sense of being alone [45]. The odds of a suicide attempt or death are reduced as the number of a person’s social connections increase, [21] highlighting the importance of social supports in reducing suicide risk. While informal supports can sometimes encourage help-seeking, [46] people experiencing high levels of distress and SI may be unwilling to disclose such thoughts to loved ones, out of concerns about being a burden or not fully understood [47]. Online peer support services have the potential to fill this gap, [48, 49] allowing people at risk of suicide to experience a person-centred connection while safely disclosing and working through their suicidal thoughts.

### Implications

Our results support the importance of using interpersonal factors to identify and assess risk of suicide more holistically and accurately. This is particularly important in the context of research that shows that about half of patients who attempt or die by suicide do not disclose suicidal thoughts [50, 51]. Clinical screening tools already in use such as K10 could potentially be augmented with brief measures of TB and PB (e.g., INQ-10) [22]. to more readily identify patients and clients with latent suicidal thinking. For services experiencing high levels of demand, such processes could be used as part of a ‘stepped care model’, a hierarchy of interventions matched to the person’s needs [52].

However, practitioners and services should be careful of approaching such assessments in a manner that is perceived by clients and patients as ‘thoughtless box-ticking’ [53]. Our findings emphasise that psychological distress and SI are linked and intensified by psychosocial factors that are intensely interpersonal: feelings of social disconnection, and perceived burden on others. This places an increased importance on the quality and strength of the therapeutic relationship which is encountered while suicidal, as this can have a significant impact on reducing thoughts of suicide [54].

Once suicidal thinking is identified, the most effective interventions focus directly on the drivers of the suicidal cognitions rather than broader symptoms of distress (Tarrier et al., 2008) [8]. A simple, effective intervention that can support the therapeutic alliance while strengthening suicide-related coping skills is the safety plan [55], a prioritised list of warning signs, coping strategies, and social supports that a person can use when suicidal thoughts intensify. Developed collaboratively, such plans have the potential to reduce suicidal thinking by naming and identifying TB and PB thinking patterns as warning signs, alongside internal coping strategies that can improve self-efficacy and counter such thinking [56]. The structured nature of a safety plan also helps practitioners and clients to easily identify gaps in their support network (e.g., lack of social supports or reasons for living) that may be exacerbating suicidal distress [57].

Our results also point to some differences in the ways TB and PB are involved in the distress/ ideation relationship. PB was more strongly involved as a mediator in the pathway, where increased distress lead to increased feeling of burdensomeness, and in turn increased SI. Increased TB was most relevant when distress was high, resulting in increased SI. As such, weakening the link between distress and feelings of burdensomeness may interrupt the pathway to SI, and have preventative benefit. Increasing belonging may be effective in clinical interventions of SI. These differences require further research.

#### Limitations and conclusions

Although the anonymous nature of the data collection utilised in this study may help to mitigate the potential of under-reporting, severity of ideation may still have been under-reported [57]. Assessments such as SIDAS which measure self-reported ideation over the past month [29] may not capture the full extent of SI when compared to momentary assessments, [58] and may not be an accurate indication of future suicide risk [57]. It must also be stated that as a cross-sectional study, causation cannot be concluded. As such, longitudinal and experimental research is needed.

The prolonged presence of suicidal thoughts is harmful to quality of life, and increases the risk of death by suicide over time. Identifying suicidal thinking patterns and their drivers early therefore forms a crucial part of suicide prevention efforts. The current study’s results shed further light on the important roles played by two motivational moderators of suicidal thinking: thwarted belongingness and perceived burdensomeness. Feelings of social disconnection and being a burden on others can take a person from a state of psychological distress into thinking about suicide, with these relationships becoming particularly strong as feelings of social disconnection increase. Brief screening tools and sensitive, person-centred approaches to explore these interpersonal factors may help to identify people at risk of suicide in situations where suicidal thoughts may not be freely disclosed. With the most effective interventions for suicide directly targeting suicidal cognitions, further research is needed to expand the range of available interventions that can address social disconnectedness and feelings of perceived burden.

## Data Availability

Data sets are available on request from the corresponding author.
